# Septic shock with no diagnosis at 24 hours: a pragmatic multicenter prospective cohort study

**DOI:** 10.1186/s13054-016-1537-5

**Published:** 2016-11-06

**Authors:** Damien Contou, Damien Roux, Sébastien Jochmans, Rémi Coudroy, Emmanuel Guérot, David Grimaldi, Sylvie Ricome, Eric Maury, Gaëtan Plantefève, Julien Mayaux, Armand Mekontso Dessap, Christian Brun-Buisson, Nicolas de Prost

**Affiliations:** 1 Service de réanimation Médicale, Groupe de Recherche CARMAS, Centre Hospitalier Universitaire Henri Mondor, Assistance Publique-Hôpitaux de Paris, 51, avenue du Maréchal de Lattre de Tassigny, Créteil, 94010 France; 2 INSERM U955, Institut Mondor de Recherche Biomedicale, Equipe 8, Faculté de Médecine de Créteil, Université Paris Est-Créteil, Créteil, France; 3 Service de réanimation médico-chirurgicale, Centre Hospitalier Universitaire Louis Mourier, Assistance Publique-Hôpitaux de Paris, 178 rue des Renouillers, Colombes, 92700 France; 4 Service de réanimation, Centre Hospitalier Marc Jacquet, 2 rue Fréteau de Peny, Melun, 77011 France; 5 Service de réanimation médicale, Centre Hospitalier Universitaire de Poitiers, 2 rue de la Milétrie, Poitiers, 86021 France; 6 Service de réanimation médicale, Centre Hospitalier Universitaire Georges Pompidou, Assistance Publique-Hôpitaux de Paris, 20 rue Leblanc, Paris, 75015 France; 7 Service de réanimation médico-chirurgicale, Centre Hospitalier André Mignot, 177 rue de Versailles, Le Chesnay, 78150 France; 8 Service de réanimation, Centre Hospitalier Robert Ballanger, Boulevard Robert Ballanger, Aulnay-sous-Bois, 93600 France; 9 Service de réanimation médicale, Centre Hospitalier Universitaire Saint-Antoine, Assistance Publique-Hôpitaux de Paris, 184 rue du Faubourg Saint-Antoine, Paris, 75012 France; 10 Service de réanimation polyvalente, Centre Hospitalier Victor Dupouy, 69 rue du Lieutenant-Colonel Prudhon, Argenteuil, 95107 France; 11 Service de réanimation médicale, Centre Hospitalier Universitaire Pitié Salpétrière, Assistance Publique-Hôpitaux de Paris, 47-83 Boulevard de l’Hôpital, Paris, 75013 France

**Keywords:** Sepsis, Septic shock, Sepsis mimickers, Intensive care, Acute mesenteric ischemia

## Abstract

**Background:**

The lack of a patent source of infection after 24 hours of management of shock considered septic is a common and disturbing scenario. We aimed to determine the prevalence and the causes of shock with no diagnosis 24 hours after its onset, and to compare the outcomes of patients with early-confirmed septic shock to those of others.

**Methods:**

We conducted a pragmatic, prospective, multicenter observational cohort study in ten intensive care units (ICU) in France. We included all consecutive patients admitted to the ICU with suspected septic shock defined by clinical suspicion of infection leading to antibiotic prescription plus acute circulatory failure requiring vasopressor support.

**Results:**

A total of 508 patients were admitted with suspected septic shock. Among them, 374 (74 %) had early-confirmed septic shock, while the 134 others (26 %) had no source of infection identified nor microbiological documentation retrieved 24 hours after shock onset. Among these, 37/134 (28 %) had late-confirmed septic shock diagnosed after 24 hours, 59/134 (44 %) had a condition mimicking septic (septic shock mimicker, mainly related to adverse drug reactions, acute mesenteric ischemia and malignancies) and 38/134 (28 %) had shock of unknown origin by the end of the ICU stay. There were no differences between patients with early-confirmed septic shock and the remainder in ICU mortality and the median duration of ICU stay, of tracheal intubation and of vasopressor support. The multivariable Cox model showed that the risk of day-60 mortality did not differ between patients with or without early-confirmed septic shock. A sensitivity analysis was performed in the subgroup (*n* = 369/508) of patients meeting the Sepsis-3 definition criteria and displayed consistent results.

**Conclusions:**

One quarter of the patients admitted in the ICU with suspected septic shock had no infection identified 24 hours after its onset and almost half of them were eventually diagnosed with a septic shock mimicker. Outcome did not differ between patients with early-confirmed septic shock and other patients.

**Electronic supplementary material:**

The online version of this article (doi:10.1186/s13054-016-1537-5) contains supplementary material, which is available to authorized users.

## Background

The lack of a patent source of infection and/or the absence of microbiological documentation is not uncommon during the first hours of management of patients presenting with a clinical phenotype of septic shock. Study of a large series of patients with sepsis in the intensive care unit (ICU) [1–3] reveals that approximately 5 % of patients with shock initially deemed to be septic had neither source of infection nor microbiological documentation retrieved by the end of the ICU stay, raising the question whether these patients truly had an underlying infection. In a monocentric study, Heffner et al. [4] showed that 18 % of patients diagnosed with sepsis had in fact a non-infectious condition that mimicked sepsis (sepsis mimicker). Two previous retrospective studies [5, 6] both showed that 13 % of the patients admitted to the ICU with suspected sepsis had in fact no infection identified; interestingly, these patients had a higher mortality rate as compared with those having an identified infection. The causes of sepsis mimickers are numerous and typically include adrenal insufficiency, acute pancreatitis, drug adverse effects, lymphoma, hemophagocytic lymphohistiocytosis or tumor lysis syndrome [4, 7].

The presence of an infectious process is by definition necessary to discriminate between sepsis and sepsis mimickers. However, patients with sepsis mimickers have a clinical phenotype that resembles that of patients with sepsis, so that antimicrobial agents are usually administered within the first hours of recognition of shock [8], until infection is ruled out and an alternative non-infectious diagnosis is made.

Managing a patient with a clinical phenotype of septic shock and with no clear diagnosis (lack of both a source of infection and microbiological documentation) within the first 24 hours of vasopressor introduction is a common but disturbing and challenging clinical scenario reflected by the often quoted question “what does my patient have?” heard during morning rounds in many ICUs. To our knowledge, the prevalence, the causes and the prognosis of suspected septic shock with no early (i.e., 24 h after vasopressor introduction) etiological diagnosis (i.e., non-early-confirmed septic shock) have never been assessed in a large series.

We conducted a pragmatic, prospective, multicenter, observational cohort study including patients initially considered to have septic shock, in which we aimed to: (1) determine the prevalence of non-early-confirmed septic shock, as defined by the lack of a definite source of infection and microbiological documentation within 24 h of shock onset; (2) identify the main causes of non-early-confirmed septic shock; and (3) compare the outcome of patients with and without early-confirmed septic shock.

## Methods

### Patients

We conducted a pragmatic, multicenter, observational, prospective cohort study in 10 ICUs in France. From November 2014 to June 2015 all consecutive patients with suspected septic shock requiring ICU admission were included. Suspected septic shock was defined by clinical suspicion of infection leading to antibiotic prescription, plus acute circulatory failure requiring vasopressor support [9] (norepinephrine or epinephrine) for more than 1 mg/h and more than 1 h. Patients with post-chemotherapy neutropenia, hemorrhagic shock, obstructive or non-septic cardiogenic shock and patients developing post-operative shock or shock post cardiac arrest were excluded. Patients with ICU-acquired septic shock (i.e., occurring more than 48 h after ICU admission) were also excluded.

Within the context of this pragmatic study reflecting real-life practice, the diagnostic work-up was not protocolized, and microbiological and imaging studies were performed at the discretion of the attending physician (see Additional file 1). Serum biomarkers, including C-reactive protein or procalcitonin were not routinely measured. In the case of lethal shock of unknown origin, an autopsy was encouraged.

### Patients’ management

All patients were promptly treated with broad-spectrum antibiotic therapy, according to the source of suspected infection, previous antibiotic treatment, and known colonization with multidrug-resistant bacteria. Source control measures, such as surgery or removal of infected devices, were applied when necessary. Patients were treated according to the 2013 guidelines of the Surviving Sepsis Campaign [8]. Low-dose steroids (hydrocortisone) were prescribed at the discretion of the clinician.

### Categorization of patients

**Fig. 1:**
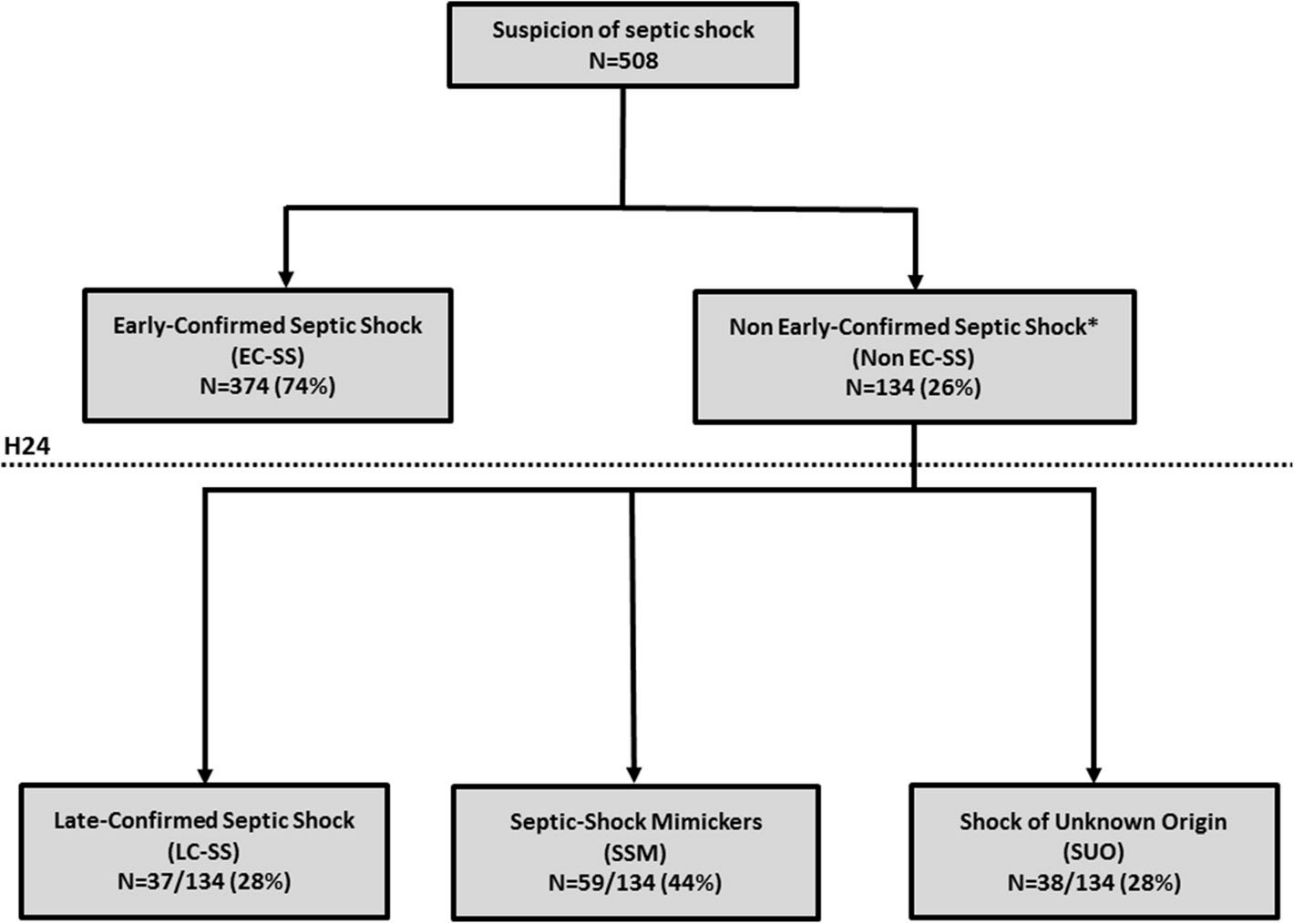
Flow chart. *Shock with no source of infection nor microbiological documentation at 24 h of study inclusion

The investigator of each center participating in the study was responsible for prospectively categorizing patients into two groups, according to data available at 24 h of inclusion: (1) patients with proven septic shock (i.e., a likely source of infection was identified by clinical or imaging studies and/or microbiological documentation was obtained within the first 24 h) were categorized as having early-confirmed septic shock (EC-SS); and (2) patients with no definite infection identified (i.e., absence of both a source of infection and microbiological documentation within the first 24 h) were categorized as having non-early-confirmed septic shock (non EC-SS) (Fig. 1).

### Definition of infection

Definitions of infection are provided in Additional file 1, and were used for the post-hoc classification of the sources of infection identified in patients with early-confirmed and late-confirmed septic shock. For all sources of infection, the absence of microbiological documentation did not *per se* exclude sepsis.

### Collection of data

Details about collected data are available in Additional file 1.

### Statistical analysis

Quantitative variables were expressed as median (25^th^–75^th^ percentiles) unless otherwise stated, and the nominal variables were reported as number (percentage). The quantitative variables were compared using the unpaired Student’s *t* test or the Mann-Whitney *U* test, and the nominal variables were compared using the chi-square (χ^2^) test or the Fisher exact test, as appropriate. The outcome of patients with EC-SS was compared to that of patients with non EC-SS. Mortality over the follow-up period was analyzed using a multivariable Cox model including variables yielding a *p* value <0.10 in univariable analysis. Potential interactions between variables introduced in the model and centers were tested using the Mantel-Haenszel test of homogeneity of odds ratios; no significant interaction was detected. Follow up was censored at the date of latest information or at 60 days, whichever occurred first. Every effort was made to obtain the post-ICU/hospital discharge vital status from investigators at each site. Survival curves were generated using the Kaplan–Meier method and compared between patients with EC-SS and non EC-SS using the log-rank test. Sensitivity analysis was performed to assess the validity of our results (i.e., proportion of patients with EC-SS and non EC-SS and Cox model for determining factors associated with mortality) in the subgroup of patients meeting septic shock criteria, as defined by the Third International Consensus Definitions for Sepsis and Septic Shock [10] (Sepsis-3). Missing data were retrieved from queries to the investigators. There was no imputation of missing data, except for data missing from comorbidities, which were then considered as absent. A two-way *p* value <0.05 was considered significant. Statistical analysis was performed using the statistical software package STATA version 13.1 (Stata Corp., College Station, TX, USA).

## Results

### One of four patients admitted in the ICU with a suspicion of septic shock had no infection identified at 24 h of shock onset

From November 2014 to June 2015, 508 patients with suspected septic shock were admitted to 10 ICUs. Among these 508 patients, 374 (74 %, 95 % CI 70–78) had EC-SS, whereas the remaining 134 (26 %, 95 % CI 22–30) lacked early confirmation (non EC-SS) (Fig. 1). There were no differences in demographic data and associated comorbidities between patients with EC-SS and patients with non EC-SS, except for diabetes mellitus, which was more frequent in the latter group (Table 1).

**Table 1:** Baseline characteristics of patients admitted to the ICU with suspected septic shock (n = 508) and comparison between patients with early-confirmed septic shocks (EC-SS) and other patients (non EC-SS)

	All patients (*n* = 508)	EC-SS (*n* = 374)	Non EC-SS (*n* = 134)	*P*
Age and gender				
Age, years	68 (59–79)	67 (58–80)	68 (61–78)	0.81
Male gender	297 (58)	219 (59)	78 (58)	>0.99
Comorbidities				
Chronic respiratory disease	21 (4)	17 (5)	4 (3)	0.44
Chronic heart failure	34 (7)	22 (6)	12 (9)	0.22
Chronic kidney disease	46 (9)	33 (9)	13 (10)	0.76
Cirrhosis	49 (10)	35 (9)	14 (10)	0.71
Recent malignant hemopathy	34 (7)	27 (7)	7 (5)	0.43
Recent cancer	63 (12)	43 (11)	20 (15)	0.30
Diabetes mellitus	132 (26)	87 (23)	45 (34)	0.019
HIV infection	17 (3)	11 (3)	6 (4)	0.40
Obesity	71 (14)	54 (14)	17 (13)	0.62
Cerebrovascular disease	55 (11)	37 (10)	18 (13)	0.26
Immunosuppressive therapy	54 (11)	41 (11)	13 (10)	0.68
Immunosuppression status^a^	132 (26)	95 (25)	37 (28)	0.62
No coexisting comorbid conditions	154 (30)	119 (32)	35 (26)	0.22
Clinical data within 24 h of inclusion and antibiotic therapy				
Maximal temperature, °C	38.2 (37.3–39.0)	38.4 (37.4 − 39.1)	37.9 (36.9 − 38.6)	<0.0001
Minimal temperature, °C	36.1 (35.2–37.0)	36.2 (35.4–37.0)	36.0 (34.6–36.8)	0.006
Glasgow Coma Scale	14 (11–15)	15 (12–15)	13 (9–15)	0.002
SAPS2	58 (47–73)	58 (45–72)	59 (50–75)	0.34
Delay admission-inclusion, hours	1 (0–6)	2 (0–7)	1 (0–4)	0.33
Delay inclusion -ATB1, hours	0 (0–1)	0 (0–1)	0 (0–1)	0.28
Delay inclusion -ATB2, hours	0 (0–2)	0 (0–2)	0 (0–2)	0.59
Number of ATB administered at 3 h	2 (1–2)	2 (1–2)	2 (1–2)	0.09
Number of ATB administered at 24 h	2 (2–3)	2 (2–3)	2 (1–3)	0.050
Biological data within 24 h of inclusion				
Leukocyte count, 10^3^.mm^−3^	16.5 (9.9–24.6)	17.1 (9.8–24.9)	15.9 (9.9–23.0)	0.62
Platelets count, 10^3^/mm^−3^	156 (85–243)	156 (81–45)	155 (92–236)	0.72
C-reactive protein, mg/L^b^	166 (87 − 265)	178 (110 − 288)	108 (48–179)	<0.0001
Procalcitonin, ng/mL^c^	7.6 (1.3–37.9)	19.6 (3.7–56.5)	1.6 (0.7–7.8)	<0.0001
Serum urea, mmol/L	14.0 (8.7–21.7)	13.6 (8.7–20.0)	16.0 (9.1–24.0)	0.16
Serum creatinine, μmoL/L	187 (114–296)	187 (115–275)	198 (107–347)	0.13
Prothrombin time, %	58 (41–72)	58 (42–72)	56 (38–72)	0.72
Arterial lactate, mmol/L	3.5 (2.0–7.0)	3.4 (2.0–6.1)	3.6 (2.0–10.0)	0.13

Categorical variables are expressed as number (%) and continuous variables as median (IQR 25–75). ^a^Immunosuppression status includes patients with HIV or malignant hemopathy or recent cancer, or under immunosuppressive therapy. ^b^Value available for 248 patients. ^c^Value available for 128 patients. *ATB* antibiotic therapy, *h* hour, *HIV* human immunodeficiency virus, *SAPS2* Simplified Acute Physiology Score, *WBC* white blood count

### Patients with non EC-SS underwent more diagnostic testing

Patients with non EC-SS underwent more imaging procedures, including computed tomography (CT) of the chest and abdomen and echocardiography, during the first 24 h of shock management, as compared to those with EC-SS (see Additional file 1: Table S1). Likewise, among the microbiological tests performed, urine, pleural and lumbar cultures were more frequently obtained in patients with non EC-SS as compared to those with EC-SS (Additional file 1: Table S1).

### Patients with non EC-SS had predominantly non-infectious disease

Only 37 (28 %) of the 134 patients with non EC-SS had infectious etiology, which was identified after a median delay of 2 (IQR 1–2) days following inclusion (late-confirmed septic shocks (LC-SS)), whereas 59 patients (44 %) had non-infectious etiology identified within 2 (1–4) days (septic shock mimickers (SSM)) and 38 patients (28 %) remained with no diagnosis by the end of the ICU stay (shock of unknown origin (SUO)) (Fig. 1 and Table 2). Causes of shock in patients with non EC-SS are shown in Table 2.

**Table 2:** Causes of shock in the 134 patients with non-early-confirmed septic shock (non EC-SS, i.e., patients with shock but no source of infection or microbiological documentation at 24 h after onset of shock)

Causes	Number (%)
Late-confirmed septic shock (LC-SS), *n*/total study sample (%)	37/134 (28)
Pleuro-pulmonary	13/37 (35)
Urinary tract	6/37 (16)
Abdomen	4/37 (11)
Liver and biliary tract	4/37 (11)
Primary bloodstream infection	4/37 (11)
Skin and soft tissues	2/37 (5)
Endocarditis	1/37 (3)
Bone-joint	1/37 (3)
Central nervous system	1/37 (3)
Endovascular stent infection	1/37 (3)
Septic shock mimickers (SSM), *n*/total study sample (%)	59/134 (44)
Adverse effects of drugs	22/59 (37)
Metformin	10
Angiotensin-converting enzyme inhibitors or angiotensin II receptor blockers	4
β-blockers	2
Propofol	2
Others^a^	4
Vascular	12/59 (20)
Acute mesenteric ischemia	12
Arterial	10
Venous	2
Malignancies	9/59 (15)
Lymphoma	3
Solid tumor (including one Marastic endocarditis)	4
Tumor lysis syndrome	2
Inflammatory diseases	5/59 (8)
Hemophagocytic lymphohistiocytosis	2
Drug rash with eosinophilia and systemic symptoms	1
Catastrophic antiphospholipid syndrome	1
Cholesterol embolization syndrome	1
Metabolic disorders	4/59 (7)
Acute adrenal insufficiency	2
Diabetic ketoacidosis	2
Acute pancreatitis	4/59 (7)
Miscellaneous	3/59 (5)
Abdominal compartment syndrome	1
Air embolism^b^	1
“Reventilation” syndrome	1
Shock of unknown origin (SUO), *n*/total study sample (%)	38/134 (28 %)

Categorical variables are expressed as n (%). ^a^Including neuroleptic (n = 1), lithium (n = 1), intravenous immunoglobulins (n = 1), sorafenib (n = 1). ^b^Air embolism was clinically suspected and confirmed by autopsy

For patients with confirmed infection (i.e., the EC-SS and LC-SS groups), the source of infection and the microorganisms isolated are reported in Additional file 1: Tables S2, S3 and S4). In both the EC-SS and LC-SS groups, the main source of infection identified was pleuro-pulmonary (Additional file 1: Table S2), including 33 % of patients (*n* = 65/195) having no microbiological documentation. Among patients (*n* = 313) who were not diagnosed with pleuro-pulmonary infection, 52 (17 %) presented with pulmonary infiltrates on chest x-ray. There was no significant difference observed between EC-SS and non EC-SS patients in the proportion of patients with leucocytes in otherwise sterile body fluids (Additional file 1: Table S5).

### Day-60 mortality was not different between EC-SS and non EC-SS patients

Patients with EC-SS developed acute respiratory distress syndrome (ARDS) more frequently thanthe other patients, although the requirement of mechanical ventilation did not differ between EC-SS and non EC-SS patients. Conversely, the non EC-SS group required renal replacement therapy more frequently (Table 3). There were no differences between these two groups in ICU mortality, duration of ICU stay and duration of mechanical ventilation or of vasopressor support (Table 3).

**Table 3:** Organ support and outcomes in patients admitted to the ICU with suspected septic shock (n = 508) and comparison between patients with early-confirmed septic shock (EC-SS) or non-early confirmed septic shock (Non EC-SS)

	All patients (*n* = 508)	EC-SS (*n* = 374)	Non EC-SS (*n* = 134)	*P*
Tracheal intubation	397 (78)	294 (79)	103 (77)	0.67
ARDS	167 (33)	143 (38)	24 (18)	<0.0001
Renal replacement therapy	145 (28)	95 (25)	50 (37)	0.009
Steroids for septic shock^a^	197 (39)	143 (38)	54 (40)	0.67
ECMO	8 (2)	5 (1)	3 (2)	0.44
Duration of tracheal intubation, days	4 (1 − 9)	4 (1 − 9)	4 (0 − 6)	0.19
Duration of vasopressors support, days	3 (2 − 5)	3 (2 − 5)	3 (2 − 6)	0.55
Duration of ICU stay, days	7 (4 − 14)	7 (4 − 24)	7 (4 − 21)	0.69
ICU mortality	188 (37)	139 (37)	49 (37)	0.90
Follow-up duration, days	29 (7 − 96)	30 (8 − 91)	25 (6 − 113)	0.75
Mortality over the follow-up period	232 (46)	166 (44)	66 (49)	0.33

Categorical variables are expressed as *n* (%) and continuous variables as median (IQR 25–75). ^a^Includes eight patients receiving either steroids or placebo because of clinical trial enrolment.

*ARDS* acute respiratory distress syndrome, *ECMO* extra-corporeal membrane oxygenation, *ICU* Intensive Care Unit

**Fig. 2:**
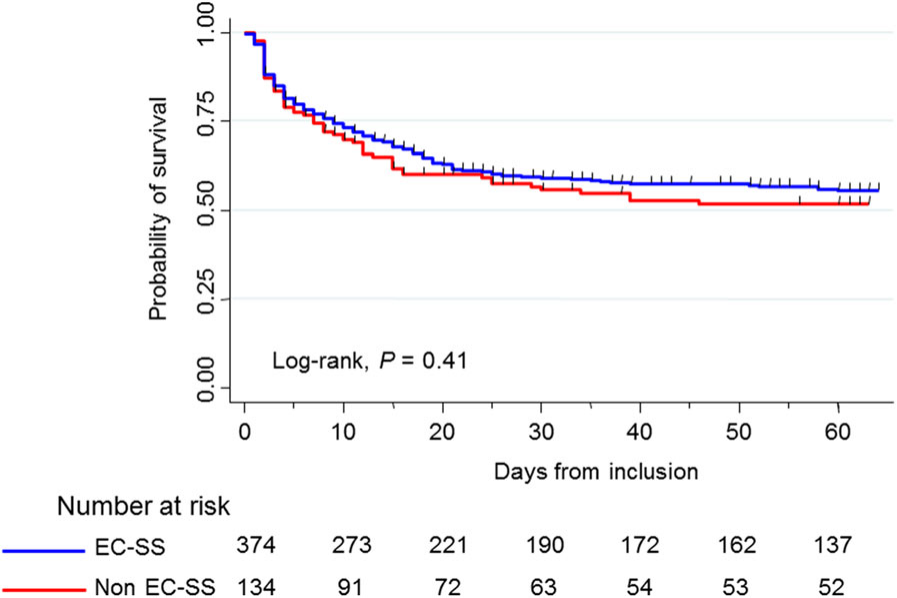
Kaplan–Meier plot of the probability of survival from inclusion to day 60 in patients with early-confirmed septic shock (*EC-SS*, *blue curve*) and other patients (*Non EC-SS*, *red curve*)

The median follow up of patients included in the cohort was 33 (6–98) days in the whole cohort and 93 (55–144) days in survivors. Among patients who survived to ICU discharge (*n* = 320), 74 had follow up of under 60 days (median 36 (26–53) days) and were censored at the time of the latest follow up. The Kaplan–Meier plot of the probability of survival from inclusion to day 60 showed no significant difference between patients with EC-SS and patients with non EC-SS (Fig. 2; *p* = 0.41, log-rank test), which was confirmed by the multivariable Cox model after adjusting for covariates associated with mortality and taking into account the center effect (Table 4). However, when patients with non EC-SS were categorized as LC-SS, SSM or SUO, those from the latter subgroup had a lower probability of survival than the others (Additional file 1: Figure S1; *p* = 0.014, log-rank test). The multivariable Cox model confirmed that patients categorized as having SUO had higher risk of mortality (Additional file 1: Table S6).

**Table 4:** Factors associated with day-60 mortality in univariable and multivariable Cox models

Variables	Univariable analysis		Multivariable analysis^a^	
	HR (95 % CI)	*P*	HR (95 % CI)	*P*
Categorization of shock				
EC-SS	1		-	-
Non EC-SS	0.87 (0.65–1.16)	0.34		
Cirrhosis				
No	1		1	
Yes	2.04 (1.42–2.93)	<0.0001	2.45 (1.63–3.69)	<0.0001
Cancer				
No	1		1	
Yes	1.62 (1.15–2.28)	0.006	1.91 (1.32–2.75)	<0.001
Chronic respiratory failure				
No	1		1	
Yes	2.16 (1.30–3.59)	0.003	2.45 (1.41–4.26)	0.002
Age >68 years				
No	1		1	
Yes	1.47 (1.14–1.91)	<0.0001	1.73 (1.30–2.30)	<0.0001
SAPS2 > 58				
No	1		1	
Yes	3.01 (2.28–3.96)	<0.0001	1.94 (1.43–2.64)	<0.0001
PT ratio <50%				
No	1		1	
Yes	1.96 (1.50–2.55)	<0.0001	1.41 (1.05–1.91)	0.024
Mechanical ventilation				
No	1		1	
Yes	3.65 (2.33–5.72)	<0.0001	2.53 (1.57–4.09)	<0.0001
Lactate, per point in mmol/L	1.09 (1.07–1.11)	<0.0001	1.06 (1.04–1.09)	<0.0001

Patients were categorized as having early-confirmed (i.e., within 24 h of vasopressor initiation) septic shock (EC-SS) or not (non EC-SS). ^a^Adjusted for center. *CI* confidence interval, *HR* hazard ratio, *PT* prothrombin time, *SAPS2* simplified acute physiology score 2

### Sensitivity analysis assessing the validity of the results in the subgroup of patients meeting the criteria of the Sepsis-3 definition

Among the 508 included patients with septic shock according to the 1992 definition [9], 369 (73 %) had serum lactate levels greater than 2 mmol/L and thus met the septic shock criteria of the Sepsis-3 definition [10]. In this subgroup, the distribution of patients with EC-SS (*n* = 272 (74 %), 95 % CI 69–78) and non EC-SS (*n* = 97 (26 %), 95 % CI 21–30) was remarkably similar with that of the whole cohort. Moreover, among patients with non EC-SS, the proportion of those with LC-SS (*n* = 23 (24 %), 95 % CI 15–32), SSM (*n* = 43 (44 %), 95 % CI 34–54), and SUO (n = 31 (32 %), 95 % CI 23–41) was also consistent with that observed in the whole cohort (Additional file 1: Table S7).

A multivariable Cox model was run in this subgroup and displayed results consistent with those in the whole cohort, with no significant relationship between non EC-SS and day-60 mortality (Additional file 1: Table S8). Also, when patients with non EC-SS were categorized as LC-SS, SSM or SUO, those from the latter subgroup had higher risk of mortality than the other patients (Additional file 1: Table S9), consistent with the results obtained in the whole cohort.

## Discussion

The main results of our pragmatic study are as follows: (1) a quarter of patients admitted to ICUs with suspected septic shock had no infection identified at 24 h after onset of shock and almost half of them had a septic shock mimicker; (2) septic shock mimickers were mostly due to acute mesenteric ischemia or adverse effects of drugs; and (3) outcomes did not differ between EC-SS and non EC-SS patients.

We reported a high rate (26 %) of patients admitted to the ICU with suspected septic shock and no clear diagnosis 24 h after its onset. This 24-h time point was defined a priori because we believed it relevant to the management of patients with sepsis syndromes, as it practically corresponds to the end of the initial management phase of critically ill patients admitted to the ICU and the time when most patients receive a probable/confirmed diagnosis. We also show that in almost three quarters of these cases (i.e., patients with SUO and SSM, accounting for 19 % of the whole cohort), sepsis was not eventually identified, despite an extensive diagnostic work-up with more imaging procedures and microbiological investigations, which is consistent with the 18 % rate reported by Heffner et al. [4] in the emergency department. Interestingly, the prevalence of diabetes mellitus was higher in patients with non EC-SS as compared to those with EC-SS, likely because of the large proportion of patients with metformin intoxication, which might also explain the higher rate of renal replacement therapy observed in this group. Conversely, the rate of ARDS was higher in patients with EC-SS, consistent with the fact that pulmonary sepsis, known to be one of the main risk factors for ARDS [11, 12], was the source of infection in half of cases in this group.

Our results thus suggest that the lack of either a source of infection or microbiological documentation, identified at 24 h after vasopressor introduction in patients admitted to and treated in the ICU on clinical suspicion of septic shock, should encourage practitioners to consider the possibility of a sepsis mimicker, which in the current study accounted for almost half of the patients in that group. In other words, more than 90 % of patients with confirmed septic shock were diagnosed within 24 h of the onset of shock and infection was secondarily confirmed in only 28 % of patients with shock having no clear diagnosis at 24 h. Our study illustrates the wide differential diagnostic spectrum of patients presenting with a clinical phenotype of septic shock.

Practically, when the etiology of a sepsis syndrome appears unclear, our series provides managing physicians with a useful working list of the main sepsis mimickers among medical admissions. The main causes of sepsis mimickers found in our study were classic [7], with a high proportion of adverse drug reactions, acute mesenteric ischemia, malignancies and inflammatory diseases.

Another important finding of the current study is that 7 % of the patients admitted to the ICU with suspected septic shock had shock of unknown origin and that both the ICU mortality and the 60-day mortality rates in these patients were higher than in other patients, including those with a septic shock mimicker. This 7 % rate is comparable to the 5 % rate of patients with no source of infection or microbiological identification reported in previous large sepsis studies [1–3], raising the question whether these patients truly had an underlying infection.

The reason for the higher risk of mortality observed in the SUO subgroup likely involves the lack of available etiological diagnosis and consequently, the impossibility of promptly initiating targeted treatment to reverse shock. In the ICU setting, autopsy studies [13–15] have been performed to assess the discrepancies between clinical and post-mortem diagnoses. Nevertheless, there is scant information in the literature on the rates of autopsy performed to identify the etiology of lethal shock. Unfortunately, none of our patients who died of shock of unknown origin underwent post-mortem imaging and only one of the three autopsies performed allowed a definite diagnosis (air embolism). Previous autopsy studies suggest that patients with lethal shock of unknown origin may have died from an undiagnosed vascular disease or acute hemorrhage, which are the main causes of missed diagnoses in the ICU setting [13]. In the context of a worldwide decline in the autopsy rate [16, 17], virtual autopsy involving post-mortem multidetector CT or magnetic resonance imaging (MRI) combined with 3D visualization may be a new alternative to medical autopsy [17, 18].

Our study has several limitations. First, the 24-h delay after onset of shock that was used to categorize patients into the EC-SS or the non EC-SS groups may be considered too short as definitive cultures of microbiological samples are not available by then. However, this delay had been defined a priori for its practical clinical relevance and eventually, only 9 % of patients with septic shock were confirmed after 24 h.

Second, patients with acute mesenteric ischemia were categorized as having SSM, which may be questionable. Nevertheless, 10 of the 12 patients diagnosed with acute mesenteric ischemia in the current series underwent surgery and had no evidence of digestive perforation or peritonitis. Moreover, all 12 patients had blood cultures drawn, none of which was positive, and 4 of 10 patients who underwent surgery had peritoneal cultures tested, and all remained sterile. Interestingly, a large recent observational study [19] showed that antibiotic therapy, although widely prescribed, is not associated with reduction of mortality in patients with acute mesenteric ischemia, thus downplaying the role of infection during acute mesenteric ischemia.

Third, a few causes of SSM may appear questionable, including ketoacidosis, reventilation syndrome or propofol-associated hypotension not meeting the diagnostic criteria for propofol infusion syndrome [20]; however, all these patients received antibiotics on clinical suspicion of sepsis and none of them had either microbiological documentation or a source of infection identified.

Fourth, one cannot rule out the possibility that some of the patients eventually diagnosed with SUO had in fact an unidentified infection [1, 21]. However, these patients had no source of infection or microbiological documentation identified by the end of the ICU hospitalization in spite of a comprehensive diagnostic work-up. We acknowledge that conventional microbiological methods frequently fail to identify a microorganism due to various reasons related to technical issues or intrinsic to the microorganism. Using polymerase chain reaction methods might have improved the early diagnosis of sepsis and helped rule out infection within 6 h of ICU admission [22, 23]. Of note, despite the limited number of patients investigated in this regard, plasma procalcitonin levels were lower in patients with SUO, suggesting the absence of an infectious process. Moreover, Combes et al. [13] previously showed that cardiovascular disease was the leading cause of missed clinical diagnoses, as opposed to infectious disease, which accounted for only 10 % of the autopsy-identified missed clinical diagnosis.

Last, the current study was designed two years before the publication of the Sepsis-3 definition [10], and the inclusion of patients was closed before its publication [10]. Thus, the quick version of the sequential organ failure assessment (SOFA) was not collected upon admission. Additionally, although all patients met the criteria for the previous definition of septic shock [9], approximately one fourth (*n* = 139) of the included patients had no hyperlactacidemia (i.e., serum lactate >2 mmol/L) upon ICU admission and would thus not have met the criteria for the Sepsis-3 definition. In any case, sensitivity analysis was performed in the subgroup of patients meeting the Sepsis-3 criteria and yielded remarkably consistent findings with those obtained from the analysis of the whole cohort.

## Conclusion

Our study showed that using conventional microbiological methods, one quarter of the patients admitted to the ICU with clinical presentation of septic shock had no infection identified 24 h after introduction of vasopressors, and almost half of these patients had a non-infectious diagnosis that mimicked sepsis. We identified several causes of septic shock mimickers for which patients with suspected septic shock of no apparent etiology should be screened. Outcomes did not differ between patients with early-confirmed septic shock and other patients. Seven percent of the patients admitted on suspicion of septic shock had no cause identified by the end of ICU stay. Further studies are needed to assess the diagnostic yield of molecular detection methods in this subgroup of patients.

## Key messages

- One quarter of the patients admitted to the ICU on suspicion of septic shock had no infection identified 24 h after onset of shock.
- Almost half of the patients with no infection identified 24 h after onset of shock had a non-infectious disease that mimicked sepsis.
- The main causes of septic shock mimickers were adverse effects of drugs, malignancies, acute mesenteric ischemia and inflammatory diseases.
- Outcomes did not differ between patients with and without early-confirmed septic shock.
- Seven percent of the patients admitted to the ICU on suspicion of septic shock had shock of unknown origin.

## Additional file

Additional file 1: Supplementary material (Table S1-Table S9 and Figure S1). (DOCX 119 kb)

## Abbreviations

ARDS: acute respiratory distress syndrome
CT: computed tomography
EC-SS: early-confirmed septic shock
ICU: intensive care unit
LC-SS: lately-confirmed septic shock
SSM: septic shock mimicker
SUO: shock of unknown origin

## References

[1] Phua J, Ngerng W, See K, Tay C, Kiong T, Lim H, Chew M, Yip H, Tan A, Khalizah H (2013). Characteristics and outcomes of culture-negative versus culture-positive severe sepsis. Crit Care.

[2] Vincent JL, Sakr Y, Sprung CL, Ranieri VM, Reinhart K, Gerlach H, Moreno R, Carlet J, Le Gall JR, Payen D (2006). Sepsis in European intensive care units: results of the SOAP study. Crit Care Med.

[3] Brun-Buisson C, Doyon F, Carlet J, Dellamonica P, Gouin F, Lepoutre A, Mercier JC, Offenstadt G, Regnier B (1995). Incidence, risk factors, and outcome of severe sepsis and septic shock in adults. A multicenter prospective study in intensive care units. French ICU Group for Severe Sepsis. JAMA.

[4] Heffner AC, Horton JM, Marchick MR, Jones AE (2010). Etiology of illness in patients with severe sepsis admitted to the hospital from the emergency department. Clin Infect Dis.

[5] Klein Klouwenberg PM, Cremer OL, van Vught LA, Ong DS, Frencken JF, Schultz MJ, Bonten MJ, van der Poll T (2015). Likelihood of infection in patients with presumed sepsis at the time of intensive care unit admission: a cohort study. Crit Care.

[6] Reyes WJ, Brimioulle S, Vincent JL (1999). Septic shock without documented infection: an uncommon entity with a high mortality. Intensive Care Med.

[7] Cohen J, Brun-Buisson C, Torres A, Jorgensen J (2004). Diagnosis of infection in sepsis: an evidence-based review. Crit Care Med.

[8] Dellinger RP, Levy MM, Rhodes A, Annane D, Gerlach H, Opal SM, Sevransky JE, Sprung CL, Douglas IS, Jaeschke R (2013). Surviving sepsis campaign: international guidelines for management of severe sepsis and septic shock: 2012. Crit Care Med.

[9] Bone RC, Balk RA, Cerra FB, Dellinger RP, Fein AM, Knaus WA, Schein RM, Sibbald WJ (1992). Definitions for sepsis and organ failure and guidelines for the use of innovative therapies in sepsis. The ACCP/SCCM Consensus Conference Committee. American College of Chest Physicians/Society of Critical Care Medicine. Chest.

[10] Singer M, Deutschman CS, Seymour CW, Shankar-Hari M, Annane D, Bauer M, Bellomo R, Bernard GR, Chiche JD, Coopersmith CM (2016). The Third International Consensus Definitions for Sepsis and Septic Shock (Sepsis-3). JAMA.

[11] Bersten AD, Edibam C, Hunt T, Moran J (2002). Incidence and mortality of acute lung injury and the acute respiratory distress syndrome in three Australian States. Am J Respir Crit Care Med.

[12] Guerin C, Reignier J, Richard JC, Beuret P, Gacouin A, Boulain T, Mercier E, Badet M, Mercat A, Baudin O (2013). Prone positioning in severe acute respiratory distress syndrome. N Engl J Med.

[13] Combes A, Mokhtari M, Couvelard A, Trouillet JL, Baudot J, Henin D, Gibert C, Chastre J (2004). Clinical and autopsy diagnoses in the intensive care unit: a prospective study. Arch Intern Med.

[14] Tejerina E, Esteban A, Fernandez-Segoviano P, Maria Rodriguez-Barbero J, Gordo F, Frutos-Vivar F, Aramburu J, Algaba A (2012). Gonzalo Salcedo Garcia O, Lorente JA. Clinical diagnoses and autopsy findings: discrepancies in critically ill patients*. Crit Care Med.

[15] Frohlich S, Ryan O, Murphy N, McCauley N, Crotty T, Ryan D (2013). Are autopsy findings still relevant to the management of critically ill patients in the modern era?. Crit Care Med.

[16] Chariot P, Witt K, Pautot V, Porcher R, Thomas G, Zafrani ES, Lemaire F (2000). Declining autopsy rate in a French hospital: physician's attitudes to the autopsy and use of autopsy material in research publications. Arch Pathol Lab Med.

[17] Roberts IS, Benamore RE, Benbow EW, Lee SH, Harris JN, Jackson A, Mallett S, Patankar T, Peebles C, Roobottom C (2012). Post-mortem imaging as an alternative to autopsy in the diagnosis of adult deaths: a validation study. Lancet.

[18] Wichmann D, Obbelode F, Vogel H, Hoepker WW, Nierhaus A, Braune S, Sauter G, Pueschel K, Kluge S (2012). Virtual autopsy as an alternative to traditional medical autopsy in the intensive care unit: a prospective cohort study. Ann Intern Med.

[19] Leone M, Bechis C, Baumstarck K, Ouattara A, Collange O, Augustin P, Annane D, Arbelot C, Asehnoune K, Baldesi O (2015). Outcome of acute mesenteric ischemia in the intensive care unit: a retrospective, multicenter study of 780 cases. Intensive Care Med.

[20] Roberts RJ, Barletta JF, Fong JJ, Schumaker G, Kuper PJ, Papadopoulos S, Yogaratnam D, Kendall E, Xamplas R, Gerlach AT (2009). Incidence of propofol-related infusion syndrome in critically ill adults: a prospective, multicenter study. Crit Care.

[21] de Prost N, Razazi K, Brun-Buisson C (2013). Unrevealing culture-negative severe sepsis. Crit Care.

[22] Bloos F, Sachse S, Kortgen A, Pletz MW, Lehmann M, Straube E, Riedemann NC, Reinhart K, Bauer M (2012). Evaluation of a polymerase chain reaction assay for pathogen detection in septic patients under routine condition: an observational study. PLoS One.

[23] Vincent JL, Brealey D, Libert N, Abidi NE, O'Dwyer M, Zacharowski K, Mikaszewska-Sokolewicz M, Schrenzel J, Simon F, Wilks M (2015). Rapid diagnosis of infection in the critically ill, a multicenter study of molecular detection in bloodstream infections, pneumonia, and sterile site infections. Crit Care Med.

