# Supplementary material for: Septic shock with no diagnosis at 24 hours: a pragmatic multicenter prospective cohort study
Source: Crit Care. 2016 Nov 6;20:360. doi: 10.1186/s13054-016-1537-5 (PMC5097846; doi:10.1186/s13054-016-1537-5)
Supplement: Supplementary file 1 — Supplementary material (Table S1-Table S9 and Figure S1). (DOCX 119 kb) [file 13054_2016_1537_MOESM1_ESM.docx]

**Septic shock with no diagnosis at 24 hours: a pragmatic multicenter prospective cohort study**

**ONLINE SUPPLEMENT**

Damien Contou, MD; Damien Roux, MD, PhD; Sébastien Jochmans, MD; Rémi Coudroy, MD; Emmanuel Guérot, MD; David Grimaldi, MD, PhD; Sylvie Ricome, MD; Eric Maury, MD, PhD; Gaëtan Plantefève, MD; Julien Mayaux, MD; Armand Mekontso Dessap, MD, PhD, Christian Brun-Buisson, MD and Nicolas de Prost, MD, PhD.

**Summary**

1. E-methods
   - Definition of infection
   - Collection of data
2. Table S1
3. Table S2
4. Table S3
5. Table S4
6. Table S5
7. Table S6
8. Table S7
9. Table S8
10. Table S9
11. Figure S1
12. References

**Definition of infection**

Bacteraemia was defined as a blood culture isolating a single recognized bacterial pathogen, except for a single sample growing coagulase-negative *Staphylococcus*, Corynebacteria or Bacillus sp. for which two positive blood cultures growing the same microorganism were required. Urinary tract infection was defined as compatible clinical and/or radiological findings and a positive urine culture (> 10^5^ cfu/mL) associated with a significant leukocyturia (> 10^4^/mL) or a clinical pyuria. Lower respiratory tract infection was defined as the association of clinical signs (cough, purulent sputum, dyspnoea, chest pain), hypoxemia, a compatible chest radiography, and/or a positive urinary antigen test or a positive quantitative culture of the respiratory tract secretions samplings. The thresholds for positivity were ≥ 10^6^, ≥ 10^3^, ≥ 10^6^ or ≥ 10^4^ cfu/mL of any single species recovered on sputum, plugged telescoping catheter, tracheal aspirate or bronchoalveolar fluid cultures, respectively. A positive culture was not mandatory to confirm a septic shock from a pulmonary origin. Abdominal infection, including biliary tract infection, diverticulitis and peritonitis was defined as the association of compatible clinical and radiological findings (computed tomography or ultrasonography) and bacterial growth from puncture or operative samplings. Soft tissue infection was clinically defined as an indurated, warm, red and painful skin area. Infection of an intra-vascular device (Canaud or implanted port catheters and pace-maker) was defined clinically (inflammation or purulence at the insertion site) or microbiologically (differential time to positivity of blood cultures higher than 2 hours or positive culture of the device). Bone/joint infections were defined as the association of compatible radiological findings (computed tomography or magnetic resonance imaging) and the growth of a pathogen from the aspirated or operative samplings.

For all sources of infection, the absence of a microbiological documentation did not *per se* exclude sepsis.

**Collection of data**

The data were prospectively collected in each ICU by experienced physicians using pre-printed case report forms. ICU discharge reports of patients with “severe SIRS” were all reviewed by two authors (DC and NdP). The following variables were recorded: (i) demographic characteristics: age, gender; (ii) date and hour of admission and inclusion; (iii) main comorbid conditions (severe chronic respiratory failure was defined by the presence of a chronic lung disease with need for home oxygen therapy, chronic heart failure by a NYHA class of 3 or 4, chronic kidney disease by a basal serum creatinine > 177μmol/L or the need for chronic hemodialysis, recent haematological malignancy or solid cancer when active or occurring in the previous year, and obesity by a body mass index higher than 30 kg.m^-2^) (iv) immunosuppressive therapy (corticosteroids > 0.5 mg/kg/d for more than 15 days, chemotherapy, methotrexate, leflunomide, cyclophosphamide, cyclosporine, tacrolimus, azathioprine, mycophenolate mofetil, rapamycine and TNF-alpha, IL-1, CD52 antagonists). Immunosuppression status included patients with HIV or haematological malignancy or recent cancer or patient receiving immunosuppressive therapy.

The administration of antibiotics within the first 24 hours was recorded, as well as their time to administration after study inclusion (*i.e.*, introduction of vasopressors).

The diagnostic work-up was recorded, including (1) Imaging studies performed such as computed tomography, ultrasonography, transoesophageal echocardiography or gastro/colonoscopies, as deemed indicated by the physician in charge; (2) Blood cultures and all other microbiological investigations obtained, including urine, ascites, cerebrospinal, synovial and pleural fluid cultures, and tests for diagnosing pneumonia, including sputum analysis, urinary antigen tests for *Streptococcus pneumoniae* and *Legionella pneumophila* and invasive respiratory tract samples (tracheal aspirate, plugged telescoping catheter or bronchoalveolar lavage), as indicated according to medical history, clinical examination and/or radiological results.

Glasgow Coma Scale and core temperature were collected and worst laboratory variables during the first 24 hours were collected including white blood cell and platelets counts, C-reactive protein and procalcitonin when obtained, blood urea nitrogen and serum creatinine, prothrombine time and arterial blood lactate. Simplified Acute Physiology Score 2 (SAPS 2) was calculated using the worst values recorded in the first 24 hours of admission [1]. The need for mechanical ventilation, renal replacement therapy, low-dose steroids and the occurrence of the acute respiratory distress syndrome [2] (ARDS) were also notified. Duration of ICU stay, in-ICU mortality and survival up to day 60 were also recorded.

**Table S1.** Diagnostic work-up performed within the first 24 hours in patients admitted in the ICU for a suspicion of septic shock (n=508) and comparison between patients with “early-confirmed” septic shock (EC-SS) and others (Non EC-SS).

|  | **All patients**  **(n=508)** | **EC-SS**  **(n=374)** | **Non EC-SS**  **(n=134)** | **p** |
| --- | --- | --- | --- | --- |
| Imaging procedures | | | | |
| Number of imaging procedures/patient | 2 [2-4] | 2 [2-4] | 3 [2-4] | <0.0001 |
| Chest X-ray | 500 (98) | 370 (99) | 130 (97) | 0.13 |
| Abdominal ultrasounds | 82 (16) | 54 (14) | 28 (21) | 0.081 |
| Renal US | 64 | 42 | 22 |  |
| Liver US | 18 | 12 | 6 |  |
| CT scan | 317 (62) | 208 (56) | 109 (81) | <0.0001 |
| Chest | 138 | 86 | 52 |  |
| Abdominal | 179 | 122 | 57 |  |
| Cerebral imaging | 67 (13) | 47 (12) | 20 (15) | 0.49 |
| CT scan | 62 | 44 | 18 |  |
| MRI | 5 | 3 | 2 |  |
| Echocardiography | 160 (31) | 91 (24) | 54 (40) | 0.0004 |
| TTE | 118 | 74 | 44 |  |
| TEE | 27 | 17 | 10 |  |
| Miscellaneous | 17 (3) | 11 (3) | 6 (4) | 0.40 |
| Digestive endoscopy | 7 | 3 | 4 |  |
| Bronchoscopy | 2 | 1 | 1 |  |
| Others | 8 | 7 | 1 |  |
| Microbiological tests | | | | |
| Blood culture | 498 (98) | 365 (98) | 133 (99) | 0.23 |
| Number drawn, mean [IQR] | 2 [1-3] | 2 [1-3] | 2 [1-2] | 0.082 |
| Urine culture | 354 (70) | 249 (67) | 105 (78) | 0.012 |
| Respiratory sample | 300 (59) | 215 (57) | 85 (63) | 0.23 |
| Sputum culture | 32 | 29 | 3 |  |
| Distal protected aspirate | 131 | 87 | 44 |  |
| BAL fluid culture | 64 | 41 | 23 |  |
| Tracheal aspirate | 152 | 118 | 34 |  |
| Urine antigen tests |  |  |  |  |
| *Streptococcus pneumoniae* | 224 (44) | 175 (47) | 49 (36) | 0.041 |
| *Legionella pneumophila* | 238 (47) | 183 (49) | 55 (41) | 0.12 |
| Ascites culture | 29 (6) | 19 (5) | 10 (7) | 0.31 |
| Pleural fluid culture | 16 (3) | 8 (2) | 8 (6) | 0.029 |
| Lumbar puncture | 52 (10) | 31 (8) | 21 (16) | 0.020 |
| Joint puncture | 8 (2) | 7 (2) | 1 (1) | 0.69 |

Categorical variables are expressed as n (%) and continuous variables as median [IQR 25-75]

***Abbreviations***

BAL: Broncho-Alveolar Lavage, CT: Computed Tomography, TEE: Trans-Esophageal Echocardiography, TTE: Trans-Thoracic Echocardiography, MRI: Magnetic Resonance Imaging, US: Ultra-Sounds

**Table S2.** Description of infection in patients with proven septic shock (n=411) and comparison between patients with “early” (EC-SS) and “lately-confirmed” septic shock (LC-SS).

|  | **All septic shock patients**  **(n=411)** | **EC-SS**  **(n=374)** | **LC-SS**  **(n=37)** | **p** |
| --- | --- | --- | --- | --- |
| **Microbiological documentation** | **320 (78)** | **289 (77)** | **31 (84)** | **0.36** |
| **BSI** | **137 (33)** | **121 (32)** | **16 (43)** | **0.18** |
| Primary BSI | 11 (3) | 7 (2) | 4 (11) | <0.001 |
| Secondary BSI^a^ | 126 (30) | 115 (30) | 12 (32) | 0.90 |
| **Effective empiric antibiotic therapy** | **301/327 (92)** | **275/296 (93)** | **26/31 (84)** | **0.085** |
| **Days from admission to microbiological documentation** | **1 [0-1]** | **0 [0-1]** | **2 [2-3]** | **<0.001** |
| **Source of infection identified** | **400 (97)** | **367 (98)** | **33 (89)** | **<0.001** |
| Pleuro-pulmonary^b^ | 195 (49) | 182 (50) | 13 (39) | - |
| Urinary tract | 64 (16) | 58 (16) | 6 (18) |  |
| Abdomen | 57 (14) | 53 (14) | 4 (12) |  |
| Skin and soft tissues | 24 (6) | 22 (6) | 2 (6) |  |
| Liver and biliary tract | 21 (5) | 17 (5) | 4 (12) |  |
| Endocarditis | 13 (3) | 12 (3) | 1 (3) |  |
| Bone-joint | 9 (2) | 8 (2) | 1 (3) |  |
| Central nervous system | 7 (2) | 6 (2) | 1 (3) |  |
| Miscellaneous^c^ | 10 (3) | 9 (2) | 1 (3) |  |

Categorical variables are expressed as n (%) and continuous variables as median [IQR 25-75]

***Abbreviations***

BSI: bloodstream infection; Primary BSI have no source identified

^a^ “secondary BSI” include urinary-tract (n=36), respiratory-tract (n=29) and intra-abdominal infections (n=15), endocarditis (n=13), biliary tract (n=10) and skin and soft tissue infections (n=9), indwelling long-term catheter-related infections (n=4), central nervous system infections (n=4) and others (n=6); ^b^ 33.3% of patients with pleuro-pulmonary infection (n=65/195; 63/182 (35%) in the EC-SS group and 2/13 (15%) in the LC-SS group) had no microbiological documentation; ^c^ including indwelling long-term catheter-related infection (n=5), endovascular stent infection (n=1), malaria (n=1), *purpura fulminans* (n=2), purulent pericarditis (n=1);

**Table S3**. Microorganisms retrieved in patients with septic shock (n=411) and comparison between patients with “early” (EC-SS) and “lately-confirmed” septic shock (LC-SS).

|  | **All septic shock patients**  **(n=411)** | **EC-SS**  **(n=374)** | **LC-SS**  **(n=37)** | **p** |
| --- | --- | --- | --- | --- |
| **Gram positive cocci^a^** | **133 (42)** | **123 (43)** | **10 (32)** | **0.47** |
| *Staphylococcus aureus* | 34 | 33 | 1 |  |
| MSSA | 31 | 30 | 1 |  |
| MRSA | 3 | 3 | 0 |  |
| Coagulase-negative *Staphylococcus* | 2 | 1 | 1 |  |
| *Streptococcus pneumoniae* | 53 | 53 | 0 |  |
| *Streptococcus* A, B, C, G | 7/3/1/0 | 6/2/1/0 | 1/1/0/0 |  |
| *Streptococcus* spp. | 18 | 16 | 2 |  |
| *Enterococcus* spp. | 10 | 9 | 1 |  |
| Unidentified CGP | 3 | 3 | 0 |  |
| **Gram negative bacilli^a^** | **161 (50)** | **147 (51)** | **14 (45)** | **0.86** |
| *Enterobacteriaceae* | 147 | 135 | 12 |  |
| *Escherichia coli*/*Proteus mirabilis* | 94/5 | 89/5 | 5/0 |  |
| Others | 48 | 41 | 7 |  |
| Unidentified GNB | 5 | 5 | 0 |  |
| *Pseudomonas aeruginosa* | 23 | 20 | 3 |  |
| Other aerobic GNB | 2 | 2 | 0 |  |
| **Other bacteria^a^** | **52 (16)** | **45 (16)** | **7 (23)** | **0.23** |
| *Haemophilus*/*Branhamella*/*Pasteurella* | 14/5/1 | 13/4/1 | 1/1/0 |  |
| *Legionella*/*Mycoplasma*/*Chlamydia* | 3/0/0 | 3/0/0 | 0/0/0 |  |
| *Neisseria meningitidis* | 2 | 2 | 0 |  |
| *Bacteroides* spp. | 6 | 4 | 2 |  |
| *Nocardia* spp. | 1 | 0 | 1 |  |
| Other anaerobic GNB | 4 | 4 | 0 |  |
| *Clostridium* spp. | 5 | 4 | 1 |  |
| Mycobacteria | 1 | 1 | 0 |  |
| **Fungi, parasites and viruses^a^** | **17 (5)** | **13 (4)** | **4 (13)** | **0.037** |
| *Candida* spp. | 6 | 4 | 2 |  |
| Other fungi | 2 | 1 | 1 |  |
| Parasite | 2 | 1 | 1 |  |
| Virus | 7 | 7 | 0 |  |
| Including pH1N1 *influenzae* | 6 | 6 | 0 |  |

Categorical variables are expressed as n (%)

***Abbreviations***

MSSA: Methicillin-Sensible *Staphylococcus aureus*, MRSA: Methicillin-resistant *Staphylococcus aureus*, CGP: Cocci-gram positive, GNB: Gram-negative bacilli

**^a^** Percentage values are reported relative to the number of microbiologically documented episodes (n=320)

Table S4. Micro-organisms isolated according to the source of infection identified

| **Source of infection identified** | **Micro-organisms isolated** | **N** |
| --- | --- | --- |
| Pleuro-pulmonary (n=195) | *Streptococcus pneumoniae* | *44* |
|  | *Haemophilus influenzae* | *22* |
|  | *Staphylococcus aureus* | *19* |
|  | Other gram positive cocci | *9* |
|  | *Enterobacteriaceae* | *32* |
|  | *Pseudomonas aeruginosa* | *10* |
|  | *Others* | *25* |
|  | No bacteria isolated | *65* |
| Urinary tract (n=64) | *Escherichia coli* | 45 |
|  | Other *Enterobacteriaceae* | 13 |
|  | *Pseudomonas aeruginosa* | 4 |
|  | Gram positive cocci | 4 |
| Abdomen (n=57) | *Escherichia coli* | 19 |
|  | Other *Enterobacteriaceae* | 7 |
|  | Other gram negative bacilli | 4 |
|  | Anaerobic bacteria | 7 |
|  | Gram positive cocci | 15 |
|  | *Clostridium difficile* | 3 |
|  | *Candida spp.* | 5 |
|  | No bacteria/fungus isolated | 17 |
| Skin and soft tissues (n=24) | Group A streptococcus | 4 |
|  | Other *Streptococcus* spp. | 5 |
|  | *Staphylococcus aureus* | 4 |
|  | *Pseudomonas aeruginosa* | 5 |
|  | Other gram negative bacilli | 4 |
|  | Others | 2 |
|  | No bacteria isolated | 4 |
| Liver and biliary tract (n=21) | *Escherichia coli* | 10 |
|  | Other *Enterobacteriaceae* | 6 |
|  | Others | 2 |
|  | No bacteria isolated | 5 |
| Endocarditis (n=13) | *Staphylococcus aureus* | 9 |
|  | Other gram positive cocci | 3 |
|  | Others | 1 |
| Bone-joint (n=9) | *Staphylococcus aureus* | 2 |
|  | Other gram positive cocci | 3 |
|  | Gram negative bacilli | 3 |
|  | No bacteria isolated | 1 |
| Central nervous system (n=7) | *Streptococcus pneumoniae* | 4 |
|  | Others | 3 |
| Miscellaneous^a^ (n=10) | *Streptococcus pneumoniae* | 2 |
|  | *Enterobacteriaceae* | 7 |
|  | Other gram positive cocci | 7 |
|  | Others | 4 |

^a^including indwelling long-term catheter-related infection (n=5), endovascular stent infection (n=1), malaria (n=1), *purpura fulminans* (n=2), purulent pericarditis (n=1)

Table S5. Presence of leucocytes in otherwise sterile body fluids

|  | **All patients**  **(n=508)** | **EC-SS**  **(n=374)** | **Non EC-SS**  **(n=134)** | **p** |
| --- | --- | --- | --- | --- |
| Urine^a^ | 130/290 (45) | 87/191 (45) | 43/99 (43) | 0.80 |
| Ascites^b^ | 4/22 (18) | 4/12 (33) | 0/10 (0) | 0.10 |
| Cerebrospinal fluid^c^ | 1/44 (2) | 1/25 (4) | 0/19 (0) | >0.99 |
| Pleural fluid, / mm^3^ | n=9  230 [40-1250] | n=5  230 [40-14155] | n=4  600 [29-1325] | >0.99 |

Categorical variables are expressed as n (%) and continuous variables as median [IQR 25-75]; ^a^ leukocyturia was defined as more than 10^4^ leucocytes /mL; ^b^ More than 250 neutrophils /mm^3^; ^c^ Meningitis was defined as more than 10 leucocytes /mm^3^

**Table S6**. Factors associated with day-60 mortality in univariable and multivariable Cox model. Patients were categorized as having an “early-confirmed” (*i.e.*, within 24 hours of vasopressors initiation) septic shock, a “lately-confirmed” septic shock, a septic shock mimicker or a shock of unknown origin.

| **Variables** | **Univariable analysis** | | **Multivariable analysis^a^** | |
| --- | --- | --- | --- | --- |
|  | HR [95% CI] | p | HR [95% CI] | p |
| **Categorization of shock**  “Early-confirmed” septic shock  “Lately-confirmed” septic shock  Septic shock mimicker  Shock of unknown origin | 1  0.81 [0.47-1.40]  0.98 [0.68-1.48]  1.91 [1.26-2.88] | 0.46  0.91  0.002 | 1  0.74 [0.41-1.33]  0.45 [0.27-0.74]  1.81 [1.14-2.88] | 0.32  0.002  0.011 |
| **Cirrhosis**  No  Yes | 1  2.04 [1.42-2.93] | <0.0001 | 1  1.83 [1.18-2.82] | 0.006 |
| **Cancer**  No  Yes | 1  1.62 [1.15-2.28] | 0.006 | 1  2.32 [1.61-3.35] | <0.0001 |
| **Chronic respiratory failure**  No  Yes | 1  2.16 [1.30-3.59] | 0.003 | 1  2.45 [1.41-4.24] | <0.001 |
| **Age > 68 years**  No  Yes | 1  1.47 [1.14-1.91] | <0.0001 | 1  1.64 [1.22-2.19] | <0.001 |
| **SAPS2 > 58**  No  Yes | 1  3.01 [2.28-3.96] | <0.0001 | 1  2.06 [1.51-2.80] | <0.0001 |
| **PT ratio < 50%**  No  Yes | 1  1.96 [1.50-2.55] | <0.0001 | 1  1.40 [1.02-1.91] | 0.038 |
| **Mechanical ventilation**  No  Yes | 1  3.65 [2.33-5.72] | <0.0001 | 1  2.74 [1.65-4.53] | 0.004 |
| **Lactate, per point in mmol/L** | 1.09 [1.07-1.11] | <0.0001 | 1.08 [1.05-1.11] | <0.0001 |

^a^ Adjusted for centres

***Abbreviations***

CI: Confidence Interval, HR: Hazard Ratio, PT: Prothrombin Time, SAPS2: Simplified Acute Physiology Score.

**Table S7.** Causes of shock in the 97 patients with a non “early-confirmed” septic shock (non EC-SS, *i.e.*, patients having a shock with no source of infection nor microbiological documentation at 24 hours of shock onset), meeting the criteria of the the Third International Definition for Sepsis and Septic Shock[3] (Sepsis 3)

| **Lately-confirmed Septic Shock (LC-SS)** | **N= 23/97 (24%)** |
| --- | --- |
| Pleuro-pulmonary | 8/23 (35) |
| Urinary tract | 4/23 (17) |
| Abdomen | 1/23 (4) |
| Liver and biliary tract | 3/23 (13) |
| Primary bloodstream infection | 4/23 (17) |
| Skin and soft tissues | 1/23 (4) |
| Bone-joint | 1/23 (4) |
| Central nervous system | 1/23 (4) |
| **Septic Shock Mimickers (SSM)** | **N=43/97 (44%)** |
| **Adverse effects of drugs** | **16/43 (37)** |
| Metformin  Angiotensin-converting enzyme inhibitors or angiotensin II receptor blockers  β-blockers  Propofol  Others^a^ | 10  2  0  1  3 |
| **Vascular** | **11/43 (26)** |
| Acute mesenteric ischemia  *Arterial*  *Venous* | 11  *9*  *2* |
| **Malignancies** | **5/43 (12)** |
| Lymphoma  Solid tumor (including one marastic endocarditis) | 1  4 |
| **Inflammatory diseases** | **1/43 (2)** |
| Hemophagocytic lymphohistiocytosis | 1 |
| **Metabolic disorders** | **4/43 (9)** |
| Acute adrenal insufficiency  Diabetic ketoacidosis | 2  2 |
| **Acute pancreatitis** | **3/43 (7)** |
| **Miscellaneous** | **3/43 (7)** |
| Abdominal compartment syndrome  Air embolism^b^  “Reventilation” syndrome | 1  1  1 |
| **Shock of Unknown Origin (SUO)** | **N=31/97 (32%)** |

Categorical variables are expressed as n (%)

^a^ Including neuroleptic (n=1), lithium (n=1) and intravenous immunoglobulins (n=1)

^b^ Air embolism was clinically suspected and confirmed by autopsy.

**Table S8**. Factors associated with day-60 mortality in univariable and multivariable Cox model among patients meeting the criteria of the Third International Definition for Sepsis and Septic Shock[3] (Sepsis-3) (n=369). Patients were categorized as having an early-confirmed (*i.e.*, within 24 hours of vasopressors initiation) septic shock (EC-SS) or not (non EC-SS).

| **Variables** | **Univariable analysis** | | **Multivariable analysis^a^** | |
| --- | --- | --- | --- | --- |
|  | HR [95% CI] | p | HR [95% CI] | p |
| **Categorization of shock**  EC-SS  Non EC-SS | 1  0.83 [0.60-1.15] | 0.27 | - | - |
| **Cirrhosis**  No  Yes | 1  1.55 [1.03-2.34] | 0.036 | 1  2.11 [1.31-3.39] | 0.002 |
| **Cancer**  No  Yes | 1  1.76 [1.19-2.61] | 0.005 | 1  1.99 [1.29-3.07] | <0.001 |
| **Chronic respiratory failure**  No  Yes | 1  1.48 [0.76-2.89] | 0.25 | - | - |
| **Age > 68 years**  No  Yes | 1  1.40 [1.04-1.88] | 0.028 | 1  1.46 [1.04-2.05] | 0.030 |
| **SAPS2 > 58**  No  Yes | 1  3.12 [2.24-4.34] | <0.0001 | 1  2.45 [1.66-3.61] | <0.0001 |
| **PT ratio < 50%**  No  Yes | 1  1.71 [1.26-2.31] | <0.0001 | - | - |
| **Mechanical ventilation**  No  Yes | 1  3.38 [2.02-5.66] | <0.0001 | 1  2.13 [1.22-3.73] | 0.008 |
| **Lactate, per point in mmol/L** | 1.09 [1.07-1.11] | <0.0001 | 1.07 [1.04-1.10] | <0.0001 |

^a^ Adjusted for centres;

***Abbreviations***

CI: Confidence Interval, HR: Hazard Ratio, PT: Prothrombin Time, SAPS2: Simplified Acute Physiology Score.

**Table S9**. Factors associated with day-60 mortality in univariable and multivariable Cox model among patients meeting the criteria of the Third International Definition for Sepsis and Septic Shock[3] (Sepsis-3) (n=369). Patients were categorized as having an “early-confirmed” (*i.e.*, within 24 hours of vasopressors initiation) septic shock, a “lately-confirmed” septic shock, a septic shock mimicker or a shock of unknown origin.

| **Variables** | **Univariable analysis** | | **Multivariable analysis^a^** | |
| --- | --- | --- | --- | --- |
|  | HR [95% CI] | p | HR [95% CI] | p |
| **Categorization of shock**  “Early-confirmed” septic shock  “Lately-confirmed” septic shock  Septic shock mimicker  Shock of unknown origin | 1  1.05 [0.57-1.94]  0.89 [0.54-1.46]  1.87 [1.19-2.94] | 0.88  0.65  0.007 | 1  0.88 [0.46-1.66]  0.31 [0.17-0.57]  1.75 [1.07-2.88] | 0.69  <0.0001  0.026 |
| **Cirrhosis**  No  Yes | 1  1.55 [1.03-2.34] | 0.036 | 1  1.84 [1.13-3.00] | 0.014 |
| **Cancer**  No  Yes | 1  1.76 [1.19-2.61] | 0.005 | 1  1.96 [1.27-3.04] | 0.003 |
| **Chronic respiratory failure**  No  Yes | 1  1.48 [0.76-2.89] | 0.25 | 1  2.00 [0.98-4.06] | 0.056 |
| **Age > 68 years**  No  Yes | 1  1.40 [1.04-1.88] | 0.028 | 1  1.46 [1.05-2.05] | 0.025 |
| **SAPS2 > 58**  No  Yes | 1  3.12 [2.24-4.34] | <0.0001 | 1  2.59 [1.76-3.81] | <0.0001 |
| **PT ratio < 50%**  No  Yes | 1  1.71 [1.26-2.31] | <0.0001 | - | - |
| **Mechanical ventilation**  No  Yes | 1  3.38 [2.02-5.66] | <0.0001 | 1  2.19 [1.25-3.83] | 0.006 |
| **Lactate, per point in mmol/L** | 1.09 [1.07-1.11] | <0.0001 | 1.10 [1.07-1.14] | <0.0001 |

^a^ Adjusted for centres

***Abbreviations***

CI: Confidence Interval, HR: Hazard Ratio, PT: Prothrombin Time, SAPS2: Simplified Acute Physiology Score.

**Figure S1.** Kaplan–Meier plot of the probability of survival from inclusion to day 60 in patients with an “early-confirmed” septic shock (EC-SS, blue curve), a « lately-confirmed » septic shock (LC-SS, black curve), a septic shock mimickers (SSM, green curve) or a shock of unknown origin (SUO, orange curve).
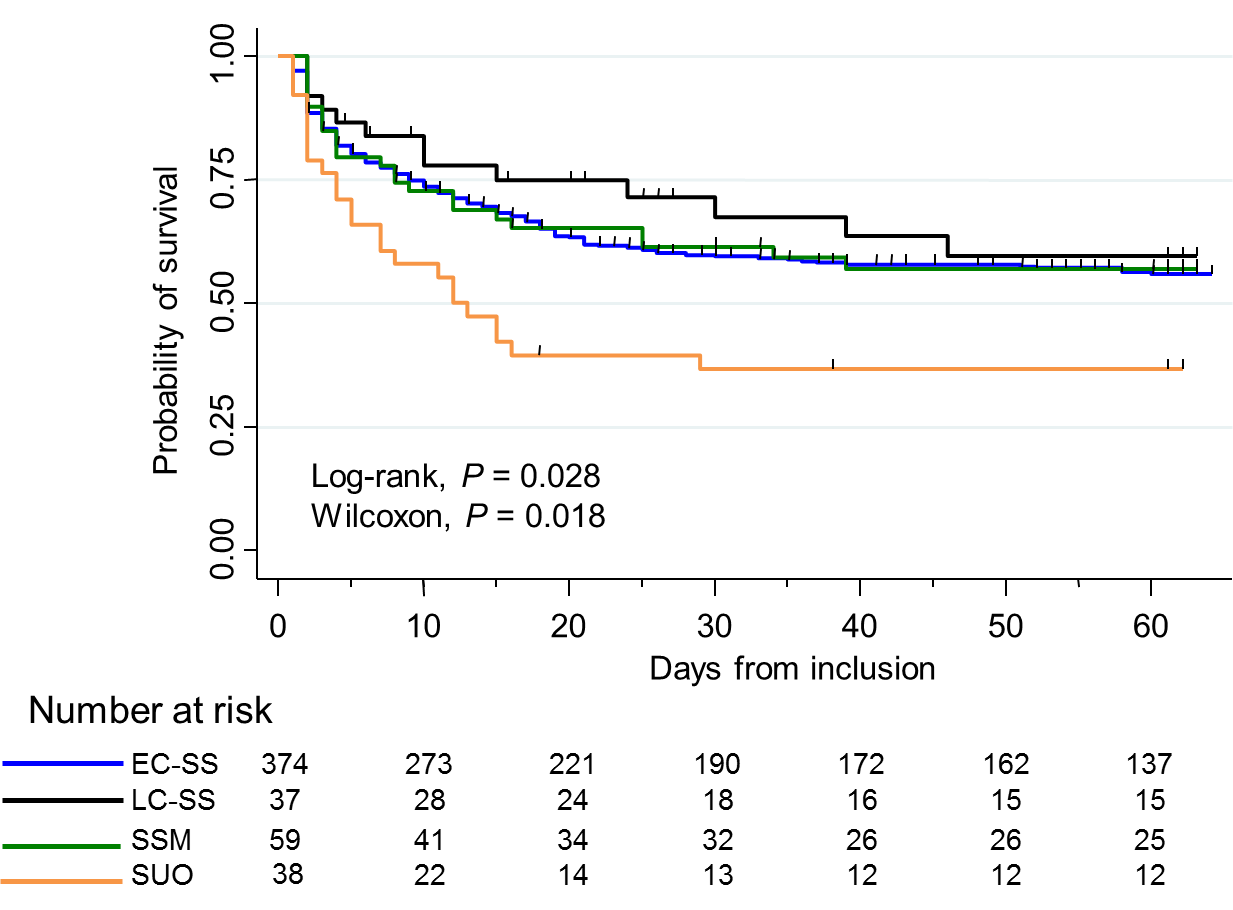


**References**

1. Le Gall JR, Lemeshow S, Saulnier F: A new Simplified Acute Physiology Score (SAPS II) based on a European/North American multicenter study. *JAMA* 1993, 270(24):2957-2963.

2. Ferguson ND, Fan E, Camporota L, Antonelli M, Anzueto A, Beale R, Brochard L, Brower R, Esteban A, Gattinoni L *et al*: The Berlin definition of ARDS: an expanded rationale, justification, and supplementary material. *Intensive Care Med* 2012, 38(10):1573-1582.

3. Singer M, Deutschman CS, Seymour CW, Shankar-Hari M, Annane D, Bauer M, Bellomo R, Bernard GR, Chiche JD, Coopersmith CM *et al*: The Third International Consensus Definitions for Sepsis and Septic Shock (Sepsis-3). *JAMA* 2016, 315(8):801-810.
